# Defining what is left in a left-sided pancreatectomy

**DOI:** 10.1093/bjs/znae096

**Published:** 2024-04-30

**Authors:** Kjetil Søreide, Ernesto Sparrelid

**Affiliations:** Division of Surgery and Oncology, Department of Clinical Science, Intervention and Technology, Karolinska Institutet, Karolinska University Hospital, Stockholm, Sweden; Department of Gastrointestinal Surgery, Hepatopancreatobiliary Unit, Stavanger University Hospital, Stavanger, Norway; Department of Clinical Medicine, University of Bergen, Bergen, Norway; Division of Surgery and Oncology, Department of Clinical Science, Intervention and Technology, Karolinska Institutet, Karolinska University Hospital, Stockholm, Sweden

Distal pancreatectomy has received relatively less attention than the eponymous Whipple operation (pancreatoduodenectomy). However, increasing attention to detail in pancreatic surgery in general, and the development of minimally invasive techniques and better appreciation of benign, premalignant, and malignant pancreatic disease entities, has changed appreciation of the details. This has resulted in the need for a more refined consideration of distal pancreatectomy—increasingly referred to as ‘left-sided pancreatectomy’. Two current studies in *BJS*^1,2^ add to the knowledge base, and come as timely additions to recently evolving evidence on optimal management in distal pancreatectomy in the Journal^3–5^.

Of note, although several aspects of open and minimally invasive surgery in distal pancreatectomy have been entertained, in addition to the role of drains^6^ and risk of postoperative fistulas^7^, several other questions remain unanswered. One is the definition of ‘left’ in left-sided pancreatectomy. Another is the question of what else can, or even should, be left (behind): the spleen? lymph nodes? peripancreatic tissue?

**Fig. 1:**
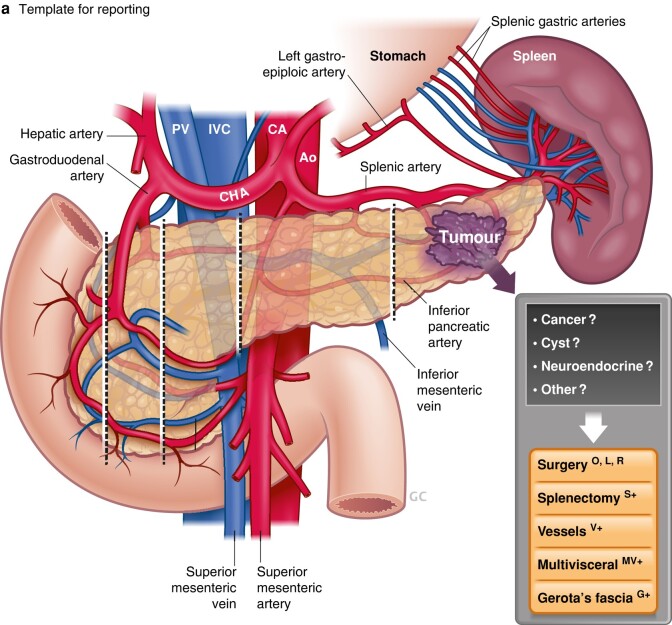

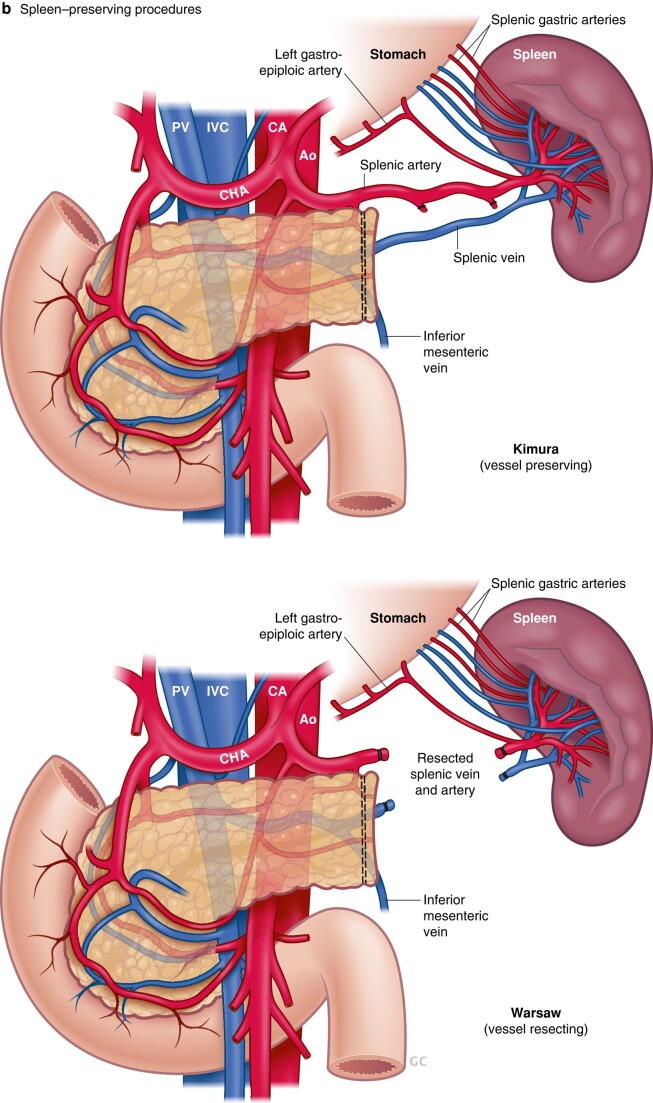

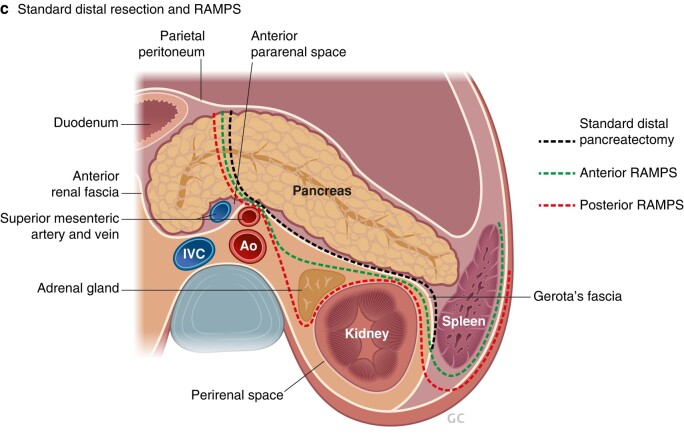
Novel terminology and definitions

Until recently, the definition of ‘left’ in left-sided pancreatectomy has been rather undeclared. In the past, the term ‘distal pancreatectomy’ was applied to any resection from a peripheral tail resection to a subtotal pancreatectomy. However, as shown in a large single-centre study^8^, there is considerable risk variation depending on the extent of resection. Hence, it is timely to see the novel definition and new terminology proposed in *BJS* by an international Delphi consensus group on left-sided pancreatectomy^1^. The new proposed terminology (*Fig. 1a*) defines the level of division of the pancreatic gland at defined anatomical landmarks, and allows a structured report of the procedure with additional parts included, such as splenectomy (designated S+) or spleen-preserving procedure (*Fig. 1b*) with vessel resection (designated V+)^1^. Multivisceral resection is designated MV+ and resection of Gerota’s fascia G+, as included in the radical antegrade modular pancreatosplenectomy (RAMPS) procedure^9^ (*Fig. 1c*), a still much debated topic. Hence, there is an opportunity to report and compare future studies that use this definition, and relate this to endpoints and assessment of outcomes.

**a** Lesion in distal pancreas, level of resection, and template for reporting. PV, portal vein; IVC, inferior vena cava; CA, coeliac artery; Ao, aorta; CHA, common hepatic artery. **b** Spleen-preserving procedures, according to Kimura or Warshaw (S+). **c** Standard distal resection; anterior radical antegrade modular pancreatosplenectomy (RAMPS), which includes resection of Gerota’s fascia (S+ G+); and posterior RAMPS, also including adrenal gland (S+ G+ MV).

Of note, the splenic vessels are not considered as part of ‘borderline’ or ‘locally advanced’ definitions in pancreatic cancer for tumours located in the body or tail^10^, even though cancers in this region also may be subject to more complex resectional procedures^11^. A lack of ‘borderline’ resectable terminology may also contribute to a lower likelihood of such tumours being considered for neoadjuvant chemotherapy, despite being associated with higher recurrence and lower survival rates^10^.

A second recurring discussion concerns the spleen, as this has commonly been considered as a part of a distal pancreatectomy, often *en passant* rather than according to indication, and with considerable variation in splenic salvage rates between institutions^12^. One reason for doing splenectomy routinely, rather than attempting a spleen-preserving procedure (*Fig. 1b*), may have been technical difficulties in dissection off the splenic vessels, particularly as practised in laparoscopic surgery. However, spleen preservation avoids the need for postsplenectomy vaccinations and the life-long increased infection risk. The introduction of robotically assisted minimally invasive surgery may facilitate spleen preservation, as demonstrated in one large study^13^ that showed higher spleen preservation rates (81% for robotic *versus* 63% laparoscopic procedures; *P* = 0.001). The spleen can be left after distal pancreatectomy by either sparing all the splenic vessels (Kimura procedure) or, even if the splenic vessels are difficult to dissect free (embedded in the pancreatic tissue, or involved by the tumour), by dividing and resecting the vessels (new terminology S+) and relying on the remaining blood flow from the gastrosplenic arteries (Warshaw procedure) (*Fig. 1b*).

**Fig. 2:**
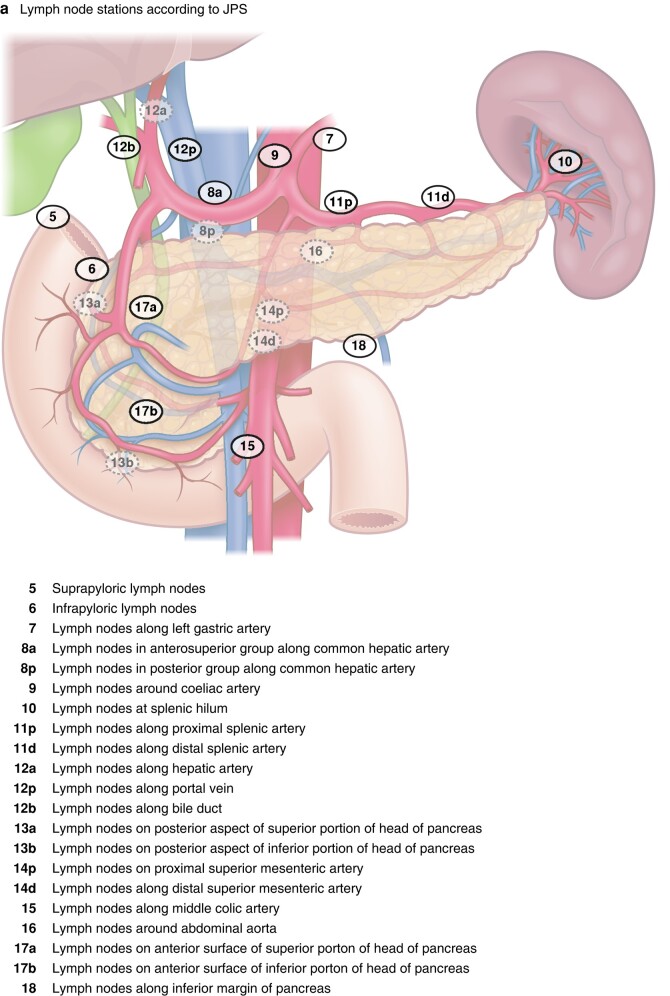

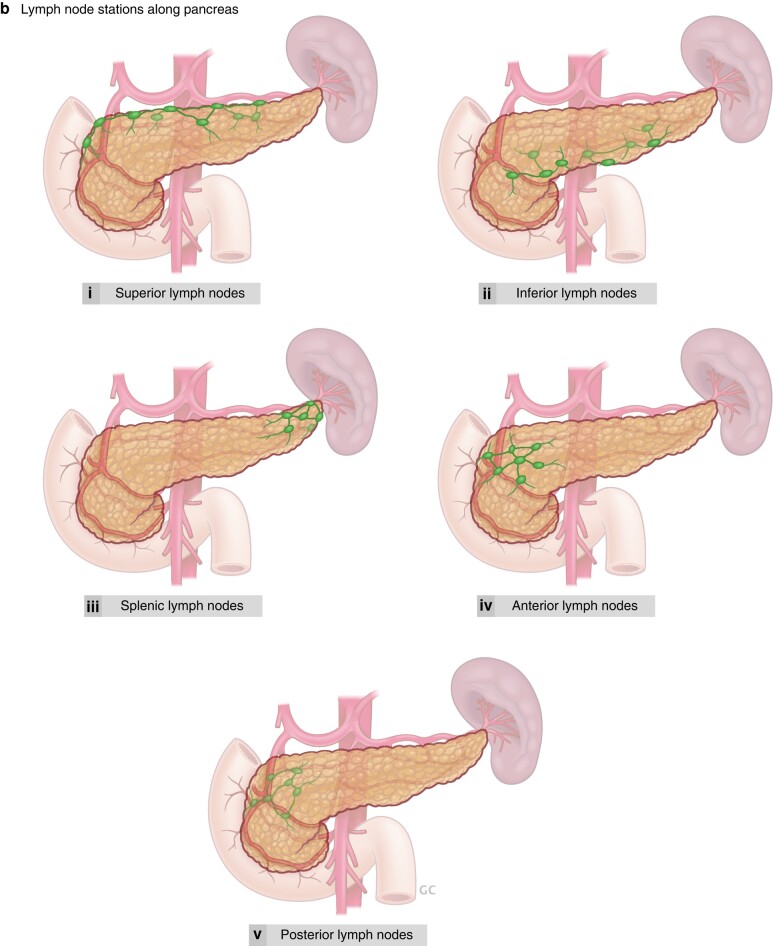
Lymph nodes of the pancreas

With an increasing number of premalignant lesions considered for surgery, in particular neuroendocrine tumour and intraductal papillary mucinous neoplasia (IPMN), the indication for splenectomy has become less clear, if not obsolete from an oncological viewpoint. Indeed, in one of the largest cohort studies^2^ to date on IPMN, less than 7% of patients had lymph nodes with metastasis in the splenic hilum. What the study does not address is the risk of having isolated lymph node metastasis in the splenic hilum alone (station 10, and hence the need for splenectomy to remove these nodes), without any other lymph node metastases along the pancreatic surface, considering the lymphatic drainage routes of the pancreas (*Fig. 2*). As nodal harvest may be viewed as a staging (and not curative) procedure, it may be considered to avoid extensive node sampling in premalignant and not clinically overt cancer diseases. Of note, this topic is bound to stir debate until further evidence can be produced to support one decision over the other. A further extended debate in this regard is the need for perirenal fat clearance (referred to as RAMPS^9^) to achieve an oncologically safe operation for advanced cancers of the body and tail of the pancreas (*Fig. 1c*).

**a** Named lymph node stations according to the proposed terminology of the Japanese Society of Pancreatology (JPS). **b** Lymph node stations along pancreatic gland as anatomically described and defined: **i** superior lymph nodes—named owing to their location along superior border of head, body, and neck of pancreas; **ii** inferior lymph nodes—named owing to their location along inferior border of head, body, and neck of pancreas; **iii** splenic lymph nodes—drain nodes long the distal part of the pancreas, hilum of the spleen, and splenorenal ligament; **iv** anterior lymph nodes—comprise pyloric, anterior pancreatoduodenal, and mesenteric lymph nodes; **v** posterior lymph nodes—include posterior pancreatoduodenal lymph nodes, which are also a common site for drainage of the (common) bile duct and hepatopancreatic ampulla (of Vater) lymphatic vessels.

Taken together, considering what is ‘left’ in left-sided pancreatectomy has now been defined by consensus^1^. To consider, tongue in cheek, what else should be left, is still a matter of debate, but with some emerging data to support decisions. The spleen may be left (spleen-preserving procedure) for benign lesions such as IPMN^2^, as the risk of metastasis to the splenic lymph nodes is low (and with uncertainty whether spread is seen isolated in this area). Further data may be accrued using the novel proposed definitions and terminology^1^, with the hope of increasing the knowledge base in decision-making for left-sided pancreatectomy.
